# Retraction Note: A natural food sweetener with anti-pancreatic cancer properties

**DOI:** 10.1038/s41389-023-00461-7

**Published:** 2023-03-16

**Authors:** C. Liu, L.-H. Dai, D.-Q. Dou, L.-Q. Ma, Y.-X. Sun

**Affiliations:** 1 Key Laboratory of Urban Agriculture (North) of Ministry of Agriculture, Beijing University of Agriculture, Beijing, China; 2 National Engineering Laboratory for Industrial Enzymes, Tianjin Institute of Industrial Biotechnology, Chinese Academy of Sciences, Tianjin, China

Retraction to: *Oncogenesis* 10.1038/oncsis.2016.28, published online 11 April 2016

The Editor-in-Chief has retracted this article because several images in this article appear to overlap with those of a previously published article [1] by different authors. In particular:

- Figure 3a (panel β-actin) appears to overlap with Figure 2e (panel 0h vs GAPDH (36 kDa)) of [1].
- Figure 3a (panel p21) appears to overlap with Figure 6b (panel APC (160 kDa)) of [1].

In addition, the authors have been unable to provide documents confirming that ethics approval was obtained for the study.

D-Q Dou agrees with this retraction. C Liu, L-H Dai, L-Q Ma and Y-X Sun have not responded to correspondence from the Publisher about this retraction.
